# Geriatric Oral Health in the United States: Current Status and Challenges

**DOI:** 10.3390/geriatrics11040084

**Published:** 2026-07-13

**Authors:** Sherif Ammar, Frederick Howard, Xi Chen, Duangporn Duangthip

**Affiliations:** 1 College of Public Health, The Ohio State University, 1841 Neil Ave, Columbus, OH 43210, USA; 2 Division of Dental Public Health, College of Dentistry, The Ohio State University, 305 W 12th Ave, Columbus, OH 43210, USA

**Keywords:** oral health, dental caries, oral disease, periodontal diseases, workforce, healthcare, public health

## Abstract

The United States is experiencing rapid population aging, making geriatric oral health an increasingly important public health and clinical concern. Older adults bear a disproportionate burden of oral diseases, including dental caries, periodontal disease, edentulism, xerostomia, and oral cancer, many of which are closely linked to chronic systemic conditions such as diabetes, cardiovascular disease, and cognitive impairment. This narrative review synthesizes current evidence on oral disease patterns and trends among older adults in the United States, with particular attention to the bidirectional relationships between oral and systemic health. It further examines the organization of oral health care delivery and financing for this population, including the roles of Medicare and Medicaid. Persistent inequities in access to preventive and restorative dental services are highlighted, especially among low-income individuals, racial and ethnic minorities, rural residents, and older adults with functional or cognitive limitations. Workforce shortages, fragmented care models, and limited integration of oral health into primary and geriatric care further exacerbate these disparities. Finally, this review identifies future directions to improve geriatric oral health, including policy reforms to expand dental coverage, integration of oral health into medical and long-term care settings, adoption of minimally invasive approaches, and strengthened interprofessional education and research. Addressing these challenges is essential to promoting healthy aging and reducing oral health disparities among older adults in the United States.

## 1. Introduction

Oral health plays a critical role in maintaining essential daily functions that support overall health, well-being and independence. The World Health Organization (WHO) defines oral health as “the state of the mouth, teeth, and orofacial structures that enables individuals to perform essential functions such as eating, breathing and speaking” [1]. Age-related declines in oral function are increasingly recognized through the concept of oral frailty, which refers to progressive deterioration of oral function, resulting from tooth loss, reduced masticatory efficiency, or impaired swallowing, ultimately leading to declines in overall health [2]. Oral health in older adults is therefore a major public health concern because this population experiences a high burden of oral disease, including periodontal diseases [3], tooth loss, and xerostomia (dry mouth) [4]. In addition, older adults commonly experience chronic systemic conditions such as cardiovascular disease, diabetes, hypertension, and cognitive decline [5,6]. Oral health is closely linked to systemic health through shared risk factors and inflammatory pathways [7]. Maintaining good oral health is thus an important component of managing chronic diseases and supporting overall health and functional independence in aging populations.

These concerns are particularly salient in the context of ongoing demographic changes in the United States (U.S.). The U.S. population aged 65 and older is projected to increase substantially from 58 million in 2022 to 82 million by 2050, representing a 42% increase. Thus, the proportion of older adults in the total population is expected to rise from 17.3% to 23% [8,9]. Women constitute the majority of this age group, accounting for 54.7% (31.9 million) of older adults, compared with 45.3% (25.9 million) of men [9]. Racial and ethnic composition within this population is also diverse, with current estimates indicating that 74.9% identify as non-Hispanic White alone, 9.4% as non-Hispanic Black alone, 4.9% as non-Hispanic Asian alone, and 9.1% as Hispanic (any race) [10].

The rapid growth of the older adult population and significant gaps in access to dental care underscore the importance of understanding whether oral health needs are being adequately addressed in the U.S. context. These gaps may exacerbate existing disparities, particularly among subgroups defined by race and ethnicity, socioeconomic status, geographic location, health behaviors such as smoking, and functional or cognitive limitations.

A recent review by Lowenstein et al. focused primarily on disparities in oral health access and outcomes among older adults [11]. However, there remains a need for a more comprehensive synthesis that also examines the organization and financing of oral health care systems as well as the growing evidence linking oral health and systemic health.

The objectives of our narrative review are to describe (1) the prevalence and patterns of major oral conditions affecting older adults; (2) the interrelationships between oral health and systemic diseases in later life; (3) financing of oral health care, including the roles of Medicare and Medicaid; (4) disparities in access to care and oral health outcomes across demographic and socioeconomic groups; and (5) system-level challenges and future directions for strengthening geriatric oral health care.

By integrating epidemiologic, clinical, and policy perspectives, this review provides a comprehensive overview of the current landscape of geriatric oral health care in the U.S. The findings are intended to inform clinicians, policymakers, and researchers and to support the development of more equitable, prevention-focused, and integrated oral health care models that promote healthy aging and reduce oral health disparities among older adults.

## 2. Methods

This review employed a narrative design. We searched PubMed/MEDLINE, Scopus, Web of Science, and Google Scholar and reviewed key U.S. federal and professional sources, including the Centers for Disease Control and Prevention, Health Resources and Services Administration, and National Institute of Dental and Craniofacial Research. The search covered January 2000 through April 2026, with more emphasis on the last 10 years to reflect current evidence and policies affecting adults aged 65 years and older. Search strategies combined controlled vocabulary (e.g., MeSH) and free-text terms related to older adults and oral health, including “older adults,” “aging,” “geriatrics,” “oral health,” “dental caries,” “periodontitis,” “tooth loss,” “edentulism,” “xerostomia,” “oral cancer,” “access to dental care,” “Medicare,” “Medicaid,” “dental workforce,” and “long-term care.” We excluded studies not focused on the United States, publications lacking data or policy-relevant content for older adults, opinion-only pieces, and duplicate or outdated versions of reports.

## 3. Oral Diseases Among Older Adults

### 3.1. Dental Caries

According to the 2017–2020 National Health and Nutrition Examination Survey (NHANES) data [12], 12.5% of adults aged 65 and older had untreated dental caries. This represents a modest decline from the 15.4% prevalence reported in the 2011–2016 cycle [13], suggesting some improvement in untreated decay among older adults. The burden of untreated decay was not evenly distributed across subgroups. Untreated decay was substantially higher among non-Hispanic Black older adults (28.4%) and Mexican American older adults (24.0%) compared with non-Hispanic White older adults (9.3%). Smoking status was associated with dental caries, with current smokers experiencing markedly higher levels of untreated decay (27.6%) than never smokers (9.7%) (Table 1).

Another analysis of NHANES 2017–2020 data examined both coronal and root caries in U.S. adults. The authors reported that coronal caries prevalence declined in older age groups, whereas root caries showed the opposite pattern, peaking among adults aged 60–69 and those 70 years and older [14]. This elevated burden of root caries in later life is largely attributed to age-related gingival recession, which exposes more root surfaces to the oral environment and increases susceptibility to decay.

### 3.2. Tooth Loss

Tooth loss patterns parallel the broader disparities in oral health. Approximately 1 in 10 adults (11.4%) aged 65–74 years had lost all their teeth or were edentulous, compared with nearly 1 in 5 adults (19.7%) aged 75 years or older [12,15]. Adults aged 75 or older had more missing teeth than those aged 65–74, with a mean tooth loss of 7.4 and 5.6 teeth, respectively. Racial and ethnic differences are pronounced. Black non-Hispanic older adults had a substantially higher prevalence of edentulism (21.8%), compared with the White non-Hispanic group (13.9%). Black non-Hispanic and Mexican American older adults had significantly fewer remaining teeth than their White non-Hispanic counterparts. Smoking status further contributes to disparities. Nearly one-third (29.4%) of the current smokers were edentulous, compared with only 11.2% of the older adults who had never smoked. Current and former smokers also had fewer remaining teeth than never-smokers. Without adjustment for confounding factors, a history of smoking was associated with tooth loss. Overall, these findings underscore the inequities in tooth loss among older adults aged 65 and older across racial, ethnic, and smoking behavioral groups. This highlights the need to strengthen prevention efforts and improve access to dental care for groups experiencing the greatest burden.

### 3.3. Periodontal Disease

NHANES classifies periodontitis according to the American Academy of Periodontology (AAP) and Centers for Disease Control and Prevention (CDC) definitions, categorizing cases into three levels: mild, moderate, and severe periodontitis (Table 2). Following the results of NHANES 2009–2012 [16], periodontitis was highly prevalent (68%) among U.S. adults aged 65 and older, and about 11% had severe periodontitis. Prevalence was higher among adults aged 75 and older (81%) than among those aged 65–74 (71.5%). Severe periodontitis was more common in men (16.2%) than in women (6.4%). Smoking emerged as one of the risk factors; current smokers had a prevalence of 92.6% for total periodontitis and 30.9% for severe periodontitis. Notably, race- and ethnicity-specific estimates are not available for adults aged 65 and older in the latest NHANES. It should be noted that no nationally representative periodontal examination has been conducted in the United States in the past ten years. As a result, outdated data limits the ability to monitor trends and assess changes in the periodontal health of the U.S older adult population over time. This highlights the need for updated national surveillance in light of demographic changes and advances in preventive care.

### 3.4. Oral Cancer

Approximately half of oral cancer cases occur among adults aged 65 and older as shown in Table 3 [17]. Using Surveillance, Epidemiology, and End Results (SEER) prevalence estimates (2019) and incidence data (2015–2019) [18,19], we examined patterns of oral cavity and pharyngeal cancer in older adults. In 2019, a total of 410,376 individuals were living with oral cavity and pharynx cancer, and most cases were in older adults: 132,426 cases among adults aged 65–74 and 115,976 cases among those aged 75 and older. Prevalence was higher in males than in females, with males showing 0.7% (ages 65–74) and 0.8% (75+) compared with 0.2% and 0.3%, respectively, among females.

Table 4 shows that incidence is higher in older adults compared with the overall population and increases further among those aged 75 years and older. Incidence rates are consistently higher in males than in females. By race and ethnicity, White adults exhibit higher incidence rates than other groups. These findings underscore the importance of integrating routine oral cancer screening into geriatric and primary care settings, particularly for populations at elevated risk.

### 3.5. Xerostomia and Medication-Related Oral Conditions

Xerostomia is a condition characterized by dry mouth, resulting from inadequate salivary flow. When the mouth becomes dry, swallowing becomes more difficult, and multiple adverse oral and systemic effects can occur. The salivary glands play a critical role in maintaining oral health [20,21]. Saliva secretion helps wash away food debris, facilitates swallowing, and protects teeth against microbial activities. When these salivary glands fail to secrete enough saliva, the mouth becomes dry. This may occur because of underlying medical conditions or as a side effect of medications and treatments that affect the salivary gland function, such as chemotherapy, immunotherapy, or radiation therapy [22,23].

Dry mouth is very common in older adults [24]. Approximately 30% of adults older than 65 years, and up to 40% of adults aged 80 or older, experience xerostomia in the US [25]. Older adults are susceptible to xerostomia because they are more likely to use medications associated with dry mouth, such as antidepressants, antihistamine drugs, or polypharmacy drugs [22,23,26]. As a result, older adults have an elevated risk of oral and dental complications. Xerostomia increases susceptibility to both coronal caries and root caries, promotes plaque accumulation, and increases gingival inflammation. Over time, these changes can contribute to the progression of periodontitis, allowing gingivitis to advance to periodontitis [26].

## 4. Oral-Systemic Links in Older Adults

Oral health and systemic health are closely interconnected in older adults. Oral diseases are highly prevalent in the elderly as a result of multimorbidity, polypharmacy, frailty, cognitive impairment, functional limitations, limited caregiver support, financial barriers, and reduced access to regular dental care [27,28,29,30]. Untreated oral disease can affect eating, reduce social interactions, and impair quality of life [31,32]. Tooth loss and oral pain may contribute to malnutrition, weight loss, and impaired immune function, accelerating functional decline and increasing morbidity and mortality [33].

### 4.1. Diabetes Mellitus and Periodontal Disease

The bidirectional relationship between periodontal disease and diabetes mellitus is one of the most well-established oral-systemic connections. Diabetes increases the risk, severity, and progression of periodontal disease through impaired neutrophil function, altered collagen metabolism, vascular changes, delayed wound healing, and exaggerated inflammatory responses within periodontal tissues [34,35,36]. Individuals with poorly controlled type 2 diabetes have a substantially increased risk and severity of periodontitis, and worsening glycemic control is associated with greater periodontal destruction and alveolar bone loss [34,36]. On the other hand, periodontal infection contributes to systemic inflammation through the release of TNF-α and IL-6, which promote insulin resistance [34]. Current evidence indicates that non-surgical periodontal therapy can result in modest but significant reductions in HbA1c in individuals with type 2 diabetes and periodontitis, highlighting the role of dental care in glycemic management [36,37,38].

### 4.2. Aspiration Pneumonia and Oral Hygiene

Poor oral hygiene increases the risk of aspiration pneumonia, a particular concern among older adults with neurogenic dysphagia, advanced frailty, or those living in institutional settings [39,40]. In the presence of poor oral hygiene, dental plaque can harbor respiratory pathogens, including Staphylococcus aureus, Gram-negative enteric bacilli, and Pseudomonas aeruginosa, which may subsequently be aspirated into the lower respiratory tract [40,41]. Evidence shows that regular tooth brushing, professional oral care, and structured oral hygiene programs can significantly reduce the incidence of aspiration pneumonia and related mortality in frail older adults and institutionalized individuals [40,42]. Daily tooth brushing has been shown to significantly reduce rates of hospital-acquired pneumonia, including meaningful reductions in both incidence and pneumonia-related mortality [43]. These findings support oral hygiene as an evidence-based preventive intervention that should be systematically implemented in acute and long-term care settings.

### 4.3. Cardiovascular Disease and Stroke

Associations between periodontal disease and cardiovascular disease have been widely reported across basic, epidemiological, and clinical studies [44,45]. Chronic periodontal inflammation may contribute to atherosclerosis through systemic dissemination of pro-inflammatory cytokines, endothelial dysfunction, oxidative stress, and transient bacteremia [44,45]. Oral microorganisms and inflammatory by-products have also been detected in atherosclerotic plaques, suggesting a plausible biological link between periodontal infection and vascular disease.

Despite strong and consistent epidemiological associations, the causal hypothesis remains under investigation. The American Heart Association acknowledges that periodontal disease is independently associated with atherosclerotic cardiovascular disease but notes that current evidence does not support a causative relationship and emphasizes the need for further studies to clarify this association [46].

### 4.4. Cognitive Impairment and Dementia

As cognitive and functional abilities decline, individuals may lose the capacity to maintain oral hygiene, recognize oral symptoms, or seek dental treatment [47,48]. As a result, older adults with dementia often experience higher rates of dental caries, periodontal disease, tooth loss, oral pain, and oral infections than their cognitively intact peers [49].

Although the mechanism remains unclear, evolving evidence suggests that the relationship between oral health and cognitive decline may be bidirectional. Poor periodontal health, including periodontitis, tooth loss, deep periodontal pockets, and alveolar bone loss, has been associated with increased risk of cognitive decline and dementia in longitudinal studies [50]. Untreated dental pain and infections may also exacerbate behavioral and psychological symptoms of dementia, including agitation, aggression, sleep disturbances, and resistance to care [51], and may further compromise quality of life [32].

### 4.5. Frailty, Nutrition, and Functional Decline

Frailty and late-life functional decline can worsen oral health through reduced mobility, diminished oral self-care ability, caregiver dependency, and limited access to dental services [52]. At the same time, tooth loss, oral pain, ill-fitting dentures, xerostomia, and reduced masticatory function can impair chewing efficiency, limit food choices, and increase swallowing difficulty, resulting in inadequate nutritional intake, weight loss, and sarcopenia [27,53]. These nutritional and functional consequences can in turn contribute to the development and progression of frailty. Studies have confirmed significant associations between oral health status and frailty in older adults, with tooth loss, periodontal disease, and impaired masticatory function each independently associated with higher frailty burden [54]. Oral health, therefore, serves not only as a marker of frailty, but also as a potentially modifiable factor influencing functional trajectories and quality of life in later life.

In sum, oral health and systemic health are closely connected in older adults: systemic disease exacerbates oral conditions, and oral disease amplifies systemic morbidity, creating compounding cycles of deterioration that are particularly damaging in the context of increased disease burden, cognitive impairment, functional limitations, and reduced physiological reserve. Interdisciplinary collaboration among dental professionals, physicians, nurses, speech-language pathologists, dietitians, caregivers, and other health care providers, together with medical–dental integration, may help reduce preventable complications, improve quality of life, and support healthier aging.

## 5. Organization and Financing of Oral Health Care

### 5.1. Oral Health Care Delivery

In the United States, older adults access oral health care through a variety of venues, including private practices, safety-net clinics, mobile and on-site programs in long-term services and supports settings, and, more recently, teledentistry and home-visit care [55]. Amid this complex suite of care, older adults are experiencing notable shifts in oral health outcomes. For over a decade, improvement in life expectancy and greater retention of natural teeth have coincided with an unchanged or increased proportion of older adults experiencing root caries and periodontal disease. As this population has grown, the absolute number of older adults living with untreated disease has risen [56,57].

While private practices and on-site clinics remain the most common means of delivery, use of teledentistry has increased markedly since the COVID-19 pandemic [58,59,60]. Delivery arrangements within long-term services and supports settings most commonly rely on mobile teams but also utilize embedded clinics or teledentistry. Across settings, persistent disparities in access and outcomes are most pronounced among low-income older adults, racial and ethnic minorities, rural residents, and individuals with other chronic illnesses [11]. Thus, an overview of oral health care delivery for older adults necessarily describes a complex network of comorbidities, pharmacological coordination, and care settings that respond unevenly to a growing population, with financing and economic organization serving as a paramount consideration.

### 5.2. Role and Limitations of Medicare in Dental Coverage

Despite the interconnected relationship between oral health, chronic disease, and pharmacological management, Medicare Parts A and B generally do not cover routine dental services such as examinations, cleanings, fillings, extractions, or dentures. In limited circumstances, dental care is covered only if other medical procedures or treatments like heart surgery or cancer treatments require oral stabilization [61]. Although many Medicare Advantage (Part C) plans advertise supplemental dental benefits, benefit structures, cost-sharing (copayments, co-insurance), and coverage for advanced treatments vary substantially across plans and regions. Consequently, benefits are often insufficient for older adults with ongoing periodontal maintenance, prosthodontic, and restorative needs [61,62].

This policy structure is increasingly misaligned with the bidirectional oral–systemic risk of older adults and the emphasis on prevention articulated by geriatric oral health experts, suggesting a need for more comprehensive dental coverage in traditional Medicare, greater integration of primary care and dentistry, and greater incentives for long-term prevention [11,58,63]. In the absence of a general, comprehensive dental benefit under Medicare, older adults must rely on out-of-pocket spending, safety net programs, or defer care altogether [58,60,64,65].

### 5.3. Role and Variability of Medicaid Dental Benefits

For low-income older adults, access to oral health care can be even more constrained. Medicaid may provide coverage for those who meet state-specific eligibility criteria; however, adult dental benefits vary widely across the United States. Differences in covered procedures, annual spending limits, definitions of medical necessity, and reimbursement levels create a geographic mosaic of access. As of 2024, 34 states provide comprehensive coverage, while 17 offer only limited or no benefits [66]. Even in states with comprehensive Medicaid benefits, only 34% of those eligible utilize these services, with rates even lower in rural areas due to a lack of dental providers [66].

States have established Medicaid eligibility thresholds between 75% and 100% of the federal poverty level (FPL), which for individuals in 2026 is $15,960 [67,68]. Research indicates that expanded benefits, broader eligibility, and increasing provider reimbursement together increase utilization [66,67,69]. Still, providers face administrative challenges, and effective patient engagement requires additional incentives and strategies that account for the economic and social instabilities of low-income living [66].

Even where comprehensive benefits are available, access still depends on provider acceptance of Medicaid and the feasibility of care delivery for home or facility-bound patients. Additionally, many states require incomes at or below 75% of FPL for Medicaid eligibility, creating what Roberts et al. call a coverage “cliff,” in which Medicare recipients with incomes modestly above the Medicaid eligibility threshold—though still within the range of FPL—face an insurmountable barrier to oral health care [70]. The result is placing contingent access and persistent inequities in prevention and treatment among the very groups at greatest risk.

### 5.4. Structural Barriers in Financing and Service Delivery

Socioeconomic status is a primary predictor of reduced access to oral health care [11]. Medicare’s limited dental coverage and Medicaid’s variable benefits contribute to disruptions in care precisely at the stage of life when oral health needs and comorbidities intensify [58]. In long-term services and supports (LTSS) facilities, staff turnover, limited staff oral care training, and resident resistance to care, especially among people living with dementia, further reduce attention to oral care and create institutional barriers [71].

Mobile clinics are frequently cited as a solution to transportation barriers; however, they face structural challenges that impact scalability and require significant technical and policy knowledge to maintain. These challenges include inconsistent state-level regulations and permitting, high financial requirements for purchasing, staffing, and maintaining specialized vehicles [72]. Similarly, teledentistry—while expanding rapidly—encounters challenges related to limited technical expertise among patients and providers, unstable internet infrastructure, poor audio-visual quality, and difficulties integrating platforms into existing administrative and reimbursement systems [73,74,75].

Recommendations to address these constraints generally follow two complementary paths. One emphasizes greater integration of dental services into primary and geriatric care visits—a simplification of the process [63]. The other calls for expanded use of interprofessional care networks in which health care providers collaborate on medication monitoring, risk assessment, and preventive planning [58,76].

## 6. Inequities in Access and Outcomes

According to the results of the National Health Interview Survey (NHIS) 2022 [77], dental visits were lower among Black adults (53.4%) compared with White non-Hispanic adults (68.1%), reinforcing the persistence of racial disparity. Socioeconomic inequities further compound these gaps. Dental visits increase steadily with income: adults with income below 100% of the federal poverty level had the lowest visit rate (35.3%), compared with 46.1% for 100–<200% FPL, 61.5% for 200–400% FPL, and 80.5% for >400% FPL [77]. Education follows the same pattern: only 33.3% of adults with middle school diploma had a dental visit, compared with 82.0% of those with a bachelor’s degree or higher. Dental coverage also plays a critical role: older adults without dental coverage had lower dental visit rates (56.4%) than those with coverage (69.6%).

Health status adds another layer of inequity. Adults with chronic diseases often face greater difficulty accessing dental care; for example, adults with diabetes had lower dental visit rates (55.1%) compared with those without diabetes (65.9%) [77]. Cognitive impairment, functional limitations, and disability may also prevent older adults from receiving needed dental care, increasing unmet oral health care needs among this population [78]. Among older adults with terminal illnesses, additional barriers include a higher disease burden, competing health care priorities, psychological distress, low perceived need for dental treatment, and a perception of limited life expectancy, all of which further prevent these individuals from accessing necessary dental care [79].

Findings from The State of Oral Health Equity in America (SOHEA) survey from CareQuest Institute further highlight gaps in dental coverage and access among older adults. Adults aged 60 and older are more likely to lack dental insurance than younger adults, and approximately one in three Medicare beneficiaries has no dental coverage. Without adequate coverage, the cost of dental care becomes a substantial burden for many seniors. CareQuest data also reveals long-standing inequities in dental utilization: one in four adults aged 65 and older has not received dental care in more than two years; racial disparities are stark—seven in ten Black seniors and six in ten Hispanic seniors have gone without dental care for over a year [80,81].

Geographic inequities further limit access to a barrier. Approximately 1 in 5 older adults living in rural areas has not seen a dental provider in five years or more. Distance to providers, limited availability of providers, and transportation challenges make routine dental care difficult to obtain, often resulting in delayed treatment until conditions become severe [81,82].

Another important contributor to oral health equality is oral health literacy, defined as the ability to obtain, understand, and use dental information to make informed oral health decisions. Evidence shows that older adults with lower health literacy are more prone to periodontal disease, have fewer remaining teeth, and experience higher levels of dental caries [83,84]. These disparities widen among those with lower educational attainment and lower socioeconomic status [85]. Moreover, caregivers of older adults with functional or cognitive limitations also share similar literacy challenges. Public health initiatives aimed at improving oral health literacy among both older adults and their caregivers have the potential to strengthen understanding of preventive strategies and increase utilization of essential preventive care [11].

Homebound and institutionalized older adults face unique barriers due to medical complexity, functional limitations, and dependence on caregivers for daily activities. These individuals often cannot travel to dental clinics and require mobile or on-site dental services. In a pilot program for homebound adults, 44% had not seen a dentist for two or more years [86]. These inequities are not a result of individual shortcomings but reflect structural vulnerability shaped by race, income, education, insurance status, disability, and geographic isolation.

Together, these factors create cumulative disadvantages that worsen oral health outcomes in later life. Addressing these inequities will require targeted policies and care models and increased investment in workforce and reimbursement structures that support access for vulnerable older populations.

## 7. System-Level Challenges

### 7.1. Workforce Shortages in Geriatric and Community-Based Dental Care

System-level gaps further hinder the integration of oral health into the broader medical system. One major challenge is workforce shortages. Even though the older adult population is rapidly growing, the oral health workforce is not expanding at a sufficient pace to meet future needs. Health Resources & Services Administration (HRSA) workforce projections (2023–2038) estimate shortages by 2038 in key oral health occupations, including about 19,860 general dentists and 33,220 dental hygienists, and the dentist shortage is expected to be about 46% in nonmetropolitan areas [87]. Geriatric dentistry is not formally recognized as a specialty in the United States, which contributes to a limited pipeline of trained providers, reduced emphasis in graduate education, and fewer incentives for clinicians to pursue careers focused on older adult populations. Addressing these inequities requires reexamining the dental workforce, including greater recognition and expansion of geriatric-focused training, as well as increased support for graduate education programs.

### 7.2. Fragmented Care Models That Separate Oral and Medical Care

A second major challenge is the fragmentation between oral and medical care models. Dental and medical services in the US are organized, financed, and delivered separately. Although oral health is closely linked to systemic health, dental care has received less attention within the health system, with limited communication, weak referral pathways, and coordination between medical and dental providers [88]. Insurance structure reinforces this divide: medical coverage has been treated as essential, while dental coverage has been optional, as seen in Medicare’s lack of routine dental benefits and variation by state in adult Medicaid dental coverage [88]. Moreover, payment structures also contribute to fragmentation. Traditional fee-for-service reimbursement prioritizes procedures over prevention, making it difficult to emphasize routine oral health and early intervention. These gaps persist across disconnected electronic health records, referral systems, and reimbursement mechanisms, all of which impede comprehensive oral care for older adults [88,89].

### 7.3. Limited Integration of Oral Health into Primary Care

Fragmentation also limits integration of oral care into primary care, geriatric care, and long-term care, despite well-established links between oral health and overall general health [90]. Gaps in workforce training in medicine are a major barrier. A national survey of U.S. geriatric fellowship programs found that 63% reported fellows receive only 1–2 h of oral health instruction, 23% reported none, and only 17% offered any clinical experience in a dental setting. The primary barriers included lack of curriculum time (57%), lack of faculty expertise (55%), and absence of national competencies (44%) [91]. As a result, many geriatric physicians graduated without adequate preparation for interprofessional dental-medical collaboration.

Integration in long-term care is also challenging. Many elderly residents depend on staff for daily oral hygiene, yet high staff turnover and limited training in nursing homes hinder the consistent delivery of routine oral care [92]. In primary care, integration is further complicated by the lack of interoperability between dental and medical electronic health records, which restricts the effective sharing of patient information [93]. Training gaps persist in this setting as well. Studies from Wisconsin and northeastern Ohio showed that although primary care physicians are willing to integrate oral health and feel comfortable with basic oral exams and referrals, they often lack the education and training needed to implement these practices in practice [94].

Experiences from other countries suggest that integrating dental care into primary care and long-term care systems is an effective approach to improving oral health in older adults. Japan offers a useful example in which oral health is increasingly incorporated into universal health and long-term care systems [95]. Dentists work more closely with physicians, nurses, and rehabilitation professionals to assess oral function, swallowing, nutrition, and frailty as part of routine geriatric care. This integrated approach helps identify and manage oral hypofunction and dysphagia earlier and positions oral health as part of overall health rather than a standalone service [54,95,96,97]. In the United Kingdom, community dental services and special care dentistry use a similar model by linking dental care with health and social care systems, particularly for older adults with complex medical or functional needs in community and residential settings [98]. Although health care structures differ, both countries highlight an important direction for the United States: greater integration of oral health into primary care, chronic disease management, geriatric assessment, and long-term care through closer interprofessional collaboration.

## 8. Coordinated Efforts to Promote Healthy Aging

Promoting healthy aging in the United States requires a coordinated, multisectoral approach that addresses the structural, clinical, and workforce barriers that shape oral health outcomes for older adults. Several strategic directions can guide national, state, and local efforts to reduce disparities and build an aging-ready oral health system.

First, policy reforms to expand dental coverage for older adults are foundational. Expanding comprehensive dental benefits through Medicare, strengthening Medicaid adult dental coverage, and incentivizing value-based payment models that reward prevention rather than procedures would reduce financial barriers and support earlier, more equitable access to care. Policy reforms should also include investments in mobile and community-based dental programs, which are essential for reaching homebound, institutionalized, and medically complex older adults.

Second, integrating oral health into medical, geriatric, and long-term care systems is critical for addressing the bidirectional relationships between oral health and chronic disease. Embedding oral health screening, risk assessment, and referral pathways into primary care workflows would enable earlier detection of disease and more coordinated management of complex medical conditions. In long-term care settings, federal and state regulations should strengthen oral health quality standards, require routine oral hygiene protocols, and ensure adequate staffing and training to support daily oral care.

Third, adopting minimally invasive and prevention-focused approaches is essential for meeting the needs of an aging population with complex medical histories. Techniques such as silver diamine fluoride and atraumatic restorative treatment offer safe, effective, and cost-efficient options for older adults who may not tolerate conventional dental procedures. Integrating these approaches into community clinics and nursing homes can reduce treatment burden, prevent disease progression, and improve quality of life. Prevention-focused care should also include nutrition counseling, xerostomia management, tobacco cessation, and caregiver-supported oral hygiene interventions.

Fourth, strengthening interprofessional education and collaborative practice is necessary to build a workforce capable of delivering integrated, age-friendly oral health care. Continuing education programs can equip health care providers with the skills needed to identify oral health problems, provide basic preventive interventions, and coordinate care across disciplines. Expanding loan repayment programs and community-based training sites can also help address dental workforce shortages in rural and underserved areas.

Finally, advancing research is essential to support healthy aging and reduce disparities. Priority areas include understanding the mechanisms linking oral health with systemic diseases; evaluating models of integrated care; and identifying strategies to reduce racial, socioeconomic, and geographic inequities [99]. In addition, strengthening surveillance systems, including NHANES and state-level oral health assessments, will provide more accurate data to guide policy and program development. Together, these future directions underscore the need for coordinated, sustained efforts across policy, clinical practice, education, and research.

These efforts align with Healthy People 2030, the current national health framework from the U.S. Department of Health and Human Services. One of its oral health objectives, increasing use of the oral health care system, is designated a Leading Health Indicator, a status given to a small number of objectives considered most important for national health. This indicator tracks dental visits among people aged 2 and older, so it covers the entire lifespan rather than focusing solely on children. Older adults are therefore fully included in this measure, even though public discourse on oral health often emphasizes pediatric care. Highlighting geriatric oral health within this framework underscores that improving access and utilization for older adults is already part of an existing federal health priority, which aligns with our narrative review and its emphasis on the unmet needs among the aging group [100,101].

This narrative review has inherent methodological limitations, as it does not follow a systematic search or standardized appraisal protocol. As a result, the synthesis may be subject to selection bias and may not capture all relevant literature on geriatric oral health care in the United States, limiting the ability to compare study quality or draw definitive conclusions. Nonetheless, the review offers important value by integrating diverse sources to provide a broad, policy-relevant overview of the challenges and opportunities in caring for an aging population. Its flexible approach allows for the inclusion of emerging themes, contextual factors, and practice-based insights that may be overlooked in more narrowly focused systematic reviews, thereby highlighting critical gaps and informing future research and policy development.

## 9. Conclusions

Older adults in the United States continue to face deep inequities in access, outcomes, and system-level support. This review highlights the disproportionate burden of oral disease among racially marginalized, low-income, rural, and medically complex older adults, driven by structural barriers that limit prevention and treatment. Building an aging-ready oral health system will require coordinated policy reforms, integrated care models, prevention-focused strategies, interprofessional training, and stronger research.

## Figures and Tables

**Table 1:** Prevalence and severity of dental caries, mean filled and retained teeth, and edentulism across adults 65–74, using National Health and Nutrition Examination Survey (NHANES 2017–2020).

Characteristic	Untreated Decay (%)	Mean Missing (MT)	Mean Filled (FT)	Mean Teeth Present	Edentulous (%)
Total	12.5	6.4	9.3	20.9	15.2
**Age**					
65–74 (Ref)	12.4	5.6	9.0	21.7	11.4
≥75	12.6	7.4 *	9.7	19.8 *	19.7 *
**Sex**					
Male (Ref)	14.0	6.5	9.0	20.7	15.7
Female	11.2	6.4	9.6	21.0	14.7
**Race and Ethnicity**					
White, non-Hispanic (Ref)	9.3	5.6	10.4	21.7	13.9
Black, non-Hispanic	28.4 *	10.6 *	4.7 *	16.2 *	21.8 *
Mexican American	24.0 *	7.6 *	7.0 *	19.7 *	15.9
**Smoking History**					
Never (Ref)	9.7	5.5	9.7	21.9	11.9
Current	27.6 *	10.4 *	7.2 *	16.3 *	29.4 *
Former	13.9	7.0 *	9.1	20.2 *	16.4

* *p* < 0.05 based on *t*-test for differences against the reference group. Estimates are from the CDC 2024 Oral Health Surveillance Report using NHANES 2017–March 2020 and represent the civilian, noninstitutionalized U.S. population aged ≥ 65 years. Untreated decay indicates one or more permanent teeth with untreated decay. MT = mean number of missing teeth due to disease; FT = mean number of filled teeth; teeth present = mean number of permanent teeth present. Edentulous indicates complete tooth loss (no natural permanent teeth). Reference groups are labeled “(Ref).” Statistically significant difference from the reference group (*p* < 0.05) as indicated in the CDC. Note: race/ethnicity categories differ by data source. NHANES/CDC reports non-Hispanic White, non-Hispanic Black, and Mexican American for comparability with previous reports; SEER reports additional racial/ethnic groups.

**Table 2:** Periodontitis prevalence and severity among U.S. adults aged ≥65 (NHANES 2009–2012) [16].

Subgroup	Mild/Moderate Periodontitis (%)	Severe Periodontitis (%)	Total Periodontitis (%)
All ≥ 65	57.0	11.0	68.0
**Age**			
65–74	59.7	11.8	71.5
≥75	71.4	9.6	81.0
**Sex**			
Male	64.1	16.2	80.2
Female	64.1	6.4	70.5
**Smoking**			
Never	64.1	9.1	73.2
Former	64.4	9.9	74.3
Current	61.8	30.9	92.6

Periodontitis case definitions follow CDC/AAP surveillance criteria. Severe: ≥2 interproximal sites with CAL ≥6 mm (not on same tooth) and ≥1 interproximal site with PPD ≥ 5 mm. Moderate: ≥2 interproximal sites with CAL ≥ 4 mm (not on same tooth) or ≥2 interproximal sites with PPD ≥ 5 mm (not on same tooth). Mild: ≥2 interproximal sites with CAL ≥ 3 mm and ≥2 interproximal sites with PPD ≥ 4 mm (not on same tooth) or ≥1 site with PPD ≥ 5 mm.

**Table 3:** Oral Cancer prevalence 2019 (SEER) by age and sex [18].

Group	All Ages (n) (%)	65–74 (n) (%)	75+ (n) (%)
All	410,376 (0.1)	132,426 (0.4)	115,976 (0.5)
Male	279,047 (0.2)	95,274 (0.7)	73,623 (0.8)
Female	131,329 (0.1)	37,152 (0.2)	42,353 (0.3)

U.S. 2019 cancer prevalence estimates are based on 2019 cancer prevalence proportions from the SEER 12 areas and the 1 January 2019 U.S. population estimates based on the average of 2018 and 2019 population estimates from the U.S. Bureau of the Census.

**Table 4:** Oral Cancer Incidence Rate (per 100,000), 2015–2019 (SEER) by Sex and Race [19].

Characteristic	All Ages	65–74 Years	75+ Years
Total (All races)	11.5	43.7	44.8
**Sex**			
Male	17.4	68.9	67.3
Female	6.4	21.9	29.0
**Race and Ethnicity**			
Non-Hispanic White	13.4	49.9	50.1
Non-Hispanic Black	8.6	31.7	27.5
Hispanic	7.1	28.1	32.4
Asian/Pacific Islander	8.6	26.8	29.5
American Indian/Alaska Native	11.0	39.4	33.3

Source: Surveillance, Epidemiology, and End Results (SEER) Program, National Cancer Institute. The 2019 cancer incidence estimates are based on 2019 cancer incidence proportions from the SEER 12 areas and the 1 January 2019 U.S. population estimates based on the average of 2018 and 2019 population estimates from the U.S. Bureau of the Census. The Alaska Native Tumor Registry only includes cases diagnosed among Alaska natives and is excluded from the analysis to avoid bias in the underlying calculations.

## Data Availability

No new data were created or analyzed in this study. Data sharing is not applicable to this article.

## Abbreviations

The following abbreviations are used in this manuscript:

U.S.	United States
WHO	World Health Organization
MeSH	Medical Subject Headings
NHANES	National Health and Nutrition Examination Survey
CDC	Centers for Disease Control and Prevention
MT	mean number of missing teeth due to disease
FT	Mean number of filled teeth
AAP	American Academy of Periodontology
CAL	clinical attachment loss
PPD	probing pocket depth
SEER	Surveillance, Epidemiology, and End Results
FPL	federal poverty level
LTSS	long-term services and supports
NHIS	National Health Interview Survey
SOHEA	State of Oral Health Equity in America
HRSA	Health Resources and Services Administration
NIDCR	National Institute of Dental and Craniofacial Research
NCHWA	National Center for Health Workforce Analysis
